# Identification and Structural-Functional Analysis of Cyclin-Dependent Kinases of the Cattle Tick *Rhipicephalus (Boophilus) microplus*

**DOI:** 10.1371/journal.pone.0076128

**Published:** 2013-10-11

**Authors:** Helga Gomes, Nelilma C. Romeiro, Gloria R. C. Braz, Eduardo Alves Gamosa de Oliveira, Camilla Rodrigues, Rodrigo Nunes da Fonseca, Naftaly Githaka, Masayoshi Isezaki, Satoru Konnai, Kazuhiko Ohashi, Itabajara da Silva Vaz, Carlos Logullo, Jorge Moraes

**Affiliations:** 1 Laboratório Integrado de Bioquímica Hatisaburo Masuda, NUPEM - UFRJ, campus Macaé, Avenida São José do Barreto, São José do Barreto, Macaé, RJ, Brazil; 2 Instituto de Bioquímica Médica, Universidade Federal do Rio de Janeiro, CCS, Bloco H, Cidade Universitária, Ilha do Fundão, Rio de Janeiro, RJ, Brazil; 3 Instituto Nacional de Ciência e Tecnologia - Entomologia Molecular, Rio de Janeiro, RJ, Brazil; 4 Departamento de Bioquímica - Instituto de Química, IQ-UFRJ, Rio de Janeiro, RJ, Brazil; 5 Centro de Biotecnologia e Faculdade de Veterinária, UFRGS, Porto Alegre, RS, Brazil; 6 Laboratório de Química e Função de Proteínas e Peptídeos, Unidade de Experimentação Animal – CBB - UENF, Horto, Campos dos Goytacazes, RJ, Brazil; 7 Laboratório Integrado de Computação Científica, NUPEM - UFRJ, Campus Macaé, São José do Barreto, Macaé, RJ, Brazil; 8 Laboratory of Infectious Diseases, Graduate School of Veterinary Medicine, Hokkaido University, Nishi, Kita-Ku Sapporo, Japan; Centro de Pesquisas René Rachou, Brazil

## Abstract

Cyclin-dependent kinases (CDKs) are a family of serine/threonine kinases essential for cell cycle progression. Herein, we describe the participation of CDKs in the physiology of *Rhipicephalus microplus*, the southern cattle tick and an important disease vector. Firstly, amino acid sequences homologous with CDKs of other organisms were identified from a *R. microplus* transcriptome database *in silico*. The analysis of the deduced amino acid sequences of CDK1 and CDK10 from *R. microplus* showed that both have caspase-3/7 cleavage motifs despite their differences in motif position and length of encoded proteins. CDK1 has two motifs (DKRGD and SAKDA) located opposite to the ATP binding site while CDK10 has only one motif (SLLDN) for caspase 3–7 near the ATP binding site. Roscovitine (Rosco), a purine derivative that inhibits CDK/cyclin complexes by binding to the catalytic domain of the CDK molecule at the ATP binding site, which prevents the transfer of ATP's γphosphoryl group to the substrate. To determine the effect of Rosco on tick CDKs, BME26 cells derived from *R. microplus* embryo cells were utilized *in vitro* inhibition assays. Cell viability decreased in the Rosco-treated groups after 24 hours of incubation in a concentration-dependent manner and this was observed up to 48 hours following incubation. To our knowledge, this is the first report on characterization of a cell cycle protein in arachnids, and the sensitivity of BME26 tick cell line to Rosco treatment suggests that CDKs are potential targets for novel drug design to control tick infestation.

## Introduction

Protein phosphorylation is a major mechanism for controlling protein activity. This leads to increased or decreased enzymatic activity or when the phosphorylation target is a transcription factor, it results in enhanced or decreased expression of the target genes. When the phosphorylation target is a regulatory protein the phosphorylation may turn on/off a metabolic pathway. The regulatory function of protein kinases has been known for several decades but the extent of this control mechanism has only been recognized fully in the recent past [1] and is now known that 5% of the proteins in any genome are eventually phosphorylated [2]. There are two major groups of protein kinases: one comprising proteins that catalyze the insertion of a phosphate group in the alcoholic hydroxyl present on the side chain of serine and threonine of the target proteins (Ser/Thr protein kinases), and another that catalyzes the insertion of a phosphate group in the phenolic hydroxyl present in the side chain of tyrosine. Some protein kinases use ATP as phosphate group donors and others use GTP or some other high-energy phosphate compounds as a phosphate source. Because of their role in signal transduction, this group of proteins has been targeted for drug design aiming to control several pathologic states, such as cancer and inflammatory diseases [3], [4].

Some proteins, like hemoglobin, have a half-life of several months [5]. However, others, including some proteins that control cell cycle division have a half-life of 3 minutes and an imperfection in cell cycle control may result in the development of cancer.

Throughout the cell cycle there are checkpoints to ensure that every step of cell division is completed correctly and that the daughter cells are identical to the mother cells. A specific group of enzymes of the family called “protein-kinases activated cyclically” play a central role in cell cycle control. The protein family responsible for activation or deactivation of those protein-kinases is known as cyclins since their concentration varies sharply during the cell cycle, with degradation occurring after the checkpoint under their control [6]. Protein kinases, which act exclusively in the presence of cyclins, are called cyclin-dependent kinases (CDKs) [7]. CDKs are the catalytic subunits of heterodimeric complexes briefly activated at specific stages of the cell cycle, and their regulation triggers the next cell cycle events [8].

Negative controllers of cell cycle act by inactivating the functions of positive controllers leading to the cell cycle arrest and apoptosis (programmed cell death) [9]. These are described as intrinsic and extrinsic negative controllers (inhibitors). The intrinsic inhibitors of CDKs are cell proteins that block the activity of CDK-cyclin complexes [10] and extrinsic CDK inhibitors are chemicals that inhibit the function of CDKs.

One of the extrinsic CDK inhibitors most often studied in cancer treatment is roscovitine (Rosco), a purine derivative that inhibits CDK1/cyclin B, CDK2/cyclin A or E, CDK5/p25, CDK7/cyclin H, and CDK9/cyclin T *in vitro* kinase assays. Rosco inhibits CDKs by binding to the catalytic domain of the CDK molecule in place of ATP, which prevents the transfer of the phosphate group to the substrate. It is commonly used as a potent antitumor drug, having high specificity to CDK, and being applied not only as a chemotherapeutic agent, but has also been described as an inhibitor of the cell cycle, when present in low concentrations [11], [12]. Ticks are the major ectoparasites of livestock and cause vast economical losses worldwide [13] by transmitting numerous pathogens to humans and animals [14]. Many tick species however have shown resistance against existing pesticides necessitating the search for more potent acaracides [15]. To our knowledge, there are no studies addressing the role of cell cycle controllers and involvement in the embryonic development in arthropod disease vectors such as the tick *Rhipicephalus microplus.* Here we present a study of the effects of roscovitine, a CDK inhibitor, on the growth and survival *in vitro* of an embryonic tick cell line (BME26), isolated from *R. microplus* embryos [16]. The focus on the CDKs from *R. microplus* is in the context of identifying suitable drug targets to control this ectoparasite at different stages of its development.

## Materials and Methods

### Identification of CDK homologs from *R. microplus*

HomoloGene (http://www.ncbi.nlm.nih.gov/homologene) is a system for automated detection of homologs among the annotated genes of several completely sequenced eukaryotic genomes. HomoloGene (release 67) was used to selectively download protein sequences of the twenty variants of CDK.

Protein sequences representatives of CDKs from Homo sapiens, Bos taurus, Mus musculus, Gallus gallus, Danio rerio, Drosophila melanogaster, Anopheles gambiae and Caenorhabditis elegans, when available, were downloaded from this database. These proteins were further used as queries to conduct BLAST searches [17] in a protein database of R. microplus (unpublished data) assembled from annotation of comprehensive R. microplus transcriptome (seven organs/tissues of R. microplus, obtained at various stages of development). The cDNA produced was sequenced by Illumina technology following the database assembly (unpublished data). In addition, the sequences obtained of R. microplus proteins were used to search for the corresponding CDK protein homologs in Ixodes scapularis, the only tick species whose genome has been sequenced [18]. A FASTA-formatted file comprising of all of the downloaded sequences, their Gene Index and accession numbers is provided (Figure S1). The sequences of R. microplus and the deduced proteins of I. scapularis are also included in this file. In some cases the sequences obtained from the transcriptome were not complete but the presence of a protein kinase domain (pfam00069) was confirmed in all cases. Eukaryotic Linear Motif Resource (http://elm.eu.org/) was used to determine the presence of the specific motifs.

### Alignment and Phylogenetic analyses

#### Classification of *R. microplus* CDKs by similarity to a restricted group of model proteins

As a first approach to confirm the classification of the nine putative CDK proteins obtained solely by blast homology with CDKs of model organisms, a phylogenetic tree was built using only CDKs found in *R. microplus*, and their best matches from *H. sapiens*, the best studied model, *B. taurus*, *R. microplus* host, and *Aedes aegypti*, an important model for vector-borne diseases. This tree was constructed by the neighbor-joining method using 5.1 MEGA software [19] and with 500 simulations to calculate bootstrap values.

#### Phylogenetic analysis of the *R. microplus* CDKs among the complete set of the 20 known CDK isoforms

A more extensive phylogenetic tree was build using Mega 5.1 software with representatives of the 20 types of CDKs from *H. sapiens, B. taurus, M. musculus, G. gallus, D. rerio, D. melanogaster, A. gambie* and *C. elegans* found in HomoloGene, together with the nine proteins deduced from transcriptome of *R. microplus* (Gene bank accession number KC968965 to KC968973) that bear sequence similarities with model CDKs. Two other proteins found during the transcriptome analysis that are important players in cell cycle progression – Nima (Never in mitosis) (KC968974) and Aurora kinase (KC968975) – were included in this analysis both to demonstrate that they are not CDKs and also to have an external group. The eight CDKs-like sequences retrieved from *I. scapularis* gene database were also included in this analysis. This phylogenetic tree was constructed as before (Figure S2).

### Molecular modeling

#### Construction of three dimensional models of CDKs from *R. microplus* by comparative modeling

Three-dimensional models of CDKs from *R. microplus* were constructed by comparative modeling using the CPH models 3.2 server (http://www.cbs.dtu.dk/services/CPHmodels/) [20], which combines sequence, structural and functional information. The template recognition is based on profile-profile alignment guided by secondary structure and exposure predictions. The accurate template determination and sequence alignment algorithm enhances the reliability of the 3D structure.

#### Models' validation

The quality of comparative models can be assessed on the basis of both geometric and energetic aspects. In this study, structural analysis (validation phase) of the CDK models from *R. microplus* was performed using the protein analysis tools available on the Structural Analysis and Verification Server (http://nihserv-110er.mbi.ucla.edu/SAVES/). Visual inspections of the three-dimensional models were made in the program PyMOL version 0.99 for Windows [21], available for download at http://www.pymol.org/.

#### Docking simulations

The structure of roscovitine was built in the software Spartan' 08 for Windows (Wavefunction, Inc.). Conformational analysis, geometry optimization and charge assignment were carried out with the semi-empirical method AM1 [22]. The CHEMSCORE_KINASE fitness function in GOLD docking software version 4.1.2 [23] was used to score the docked compounds. The active site was defined as all atoms within 10 Å from O768 from Val140, in CDK1 and O810 from Cys147 in CDK10 models, respectively, which have been used as reference residues for binding site definition in GOLD software. The protein-ligand complexes with the most favorable Fitness score values among the top scored complexes were used for further visual inspection, which has been done with Pymol v. 0.99 [21].

### Cloning of CDK1 and CDK10 from *R. microplus* ovary

RNA was isolated from ovaries of fully engorged female *R. microplus* (Porto Alegre isolate) ticks using TRIZOL reagent according to the manufacturers' instructions. Complementary DNA (cDNA) was subsequently synthesized from total RNA by using Superscript® III reverse transcriptase and *oligo* (*dT*) *12–18* primer. PCR were performed using specific primers designed from *R. microplus* CDK sequences retrieved from the transcriptome database.

The primers used were FOR-1 (5′- GGGGGCAGTCGAAGCGG -3′) which corresponds to the 5′ end, and REV-1 (5′- AAATACTGATGTTTGAGG -3′), which corresponds to the 3′ end of Rm-CDK1 and FOR-10 (5′- ATGGAAGCCGGCATAAATCAAC -3′) which corresponds to the 5′ end, and REV-10 (5′- TCAGATGAAGTCTGCAGTCT -3′), which corresponds to the 3′ end of Rm-CDK10. The amplicons were purified on agarose gels and ligated into the pGEM-T vector (Promega), and transformed into DH5α strain of *Escherichia coli*. The cloned insert were sequenced on a P/ACE™ MDQ-Beckman Coulter, Inc. Automated sequencer.

### BME26 cell line

Cells were maintained following a previously described protocol [16]. Briefly, adherent cells from 25 cm^2^ confluent flasks were suspended into a fresh complete medium using a 22-gauge needle with a bent tip fitted to a plastic syringe. Cells were passaged every 3–4 weeks, and the medium replaced weekly. Culture density was determined with a Neubauer hemocytometer and cell viability was determined by the trypan blue exclusion (0.4%) method. Two weeks prior to their use in assays, the synchronized cells were prepared by seeding 1×10^7^ cells into 5 mL of fresh complete medium (final volume), and growing them at 34°C to ensure doubling (within 2 weeks), finally the medium was replaced weekly.

### Viability assay

BME26 cell suspension was seeded into 24-well plates at a density of 5×10^5^ cells/well, to a final volume of 500 µL of complete medium and allowed to attach. After 24 h at 34°C, roscovitine was added at the final concentrations indicated (75 µM, 150 µM, 175 µM, 200 µM or 225 µM), and 0.1% DMSO was used in negative control wells. After 24 or 48 h of treatment, 50 µL of tetrazolium salt MTT prepared in serum-free medium (5 mg/mL) was added to each well. After additional 2 h incubation, the media was completely discarded and 1 mL of acid-isopropyl alcohol (0.15% HCl in isopropyl alcohol) was added to dissolve the formazan crystals. The mixture was transferred to 1.5 mL tubes, spun at 6,000×g for 15 min, and the clear supernatant collected in new tubes for absorbance measurement at 570 nm using quartz cuvettes in an UVmini-1240 UV-VIS spectrophotometer (Shimadzu, Japan). Unless otherwise stated, absorbance values of control treatment were used for normalization (100% viability).

### BME26 cell staining with hematoxylin-eosin staining (HE)

BME26 cells were plated (5×10^5^ cell/well) over glass coverslips placed at the bottom of a 24-well plate to a final volume of 500 µL of complete medium and allowed to attach. After 24 h at 34°C, roscovitine was added at the final concentrations indicated (75 µM; 150 µM; 175 µM; 200 µM; 225 µM), and 0.1% DMSO was used in negative control wells. After 24 h of treatment, the coverslips were removed from the plates and the staining was performed [24]. Image J software was used to quantify the number of cells per field in each treatment. Three independent experiments for each treatment were performed in triplicates and every replicate analyzed under 3 different fields with image J.

### Transcription of Rm-CDK in BME26

The transcription of Rm-CDKs in BME26 cell was evaluated by RT-PCR. Total RNA was extracted from cells using TRIzol reagent (Invitrogen) following the manufacturer's recommendations. One microgram of total RNA reverse-transcribed using the Reverse Transcriptase M-MLV (Takara) and oligo(dT) primer. Specific primers designed for gene amplifications are described in Table S1. The reactions were performed according to the following steps: 1 min at 94°C followed by 35 cycles of 1 min at 94°C, 30 s at 55°C, and 1 min at 72°C, with a final elongation at 72°C for 7 min. PCR products were electrophoresed on a 2% agarose gel and visualized by staining with ethidium bromide. The elongation factor 1α (ELF1A) gene primers were used as positive control [25].

## Results

### Identification of putative *R. microplus* CDKs and selection of Rm-CDK1 and Rm-CDK10 isoforms for further analyses

**Figure 1:**
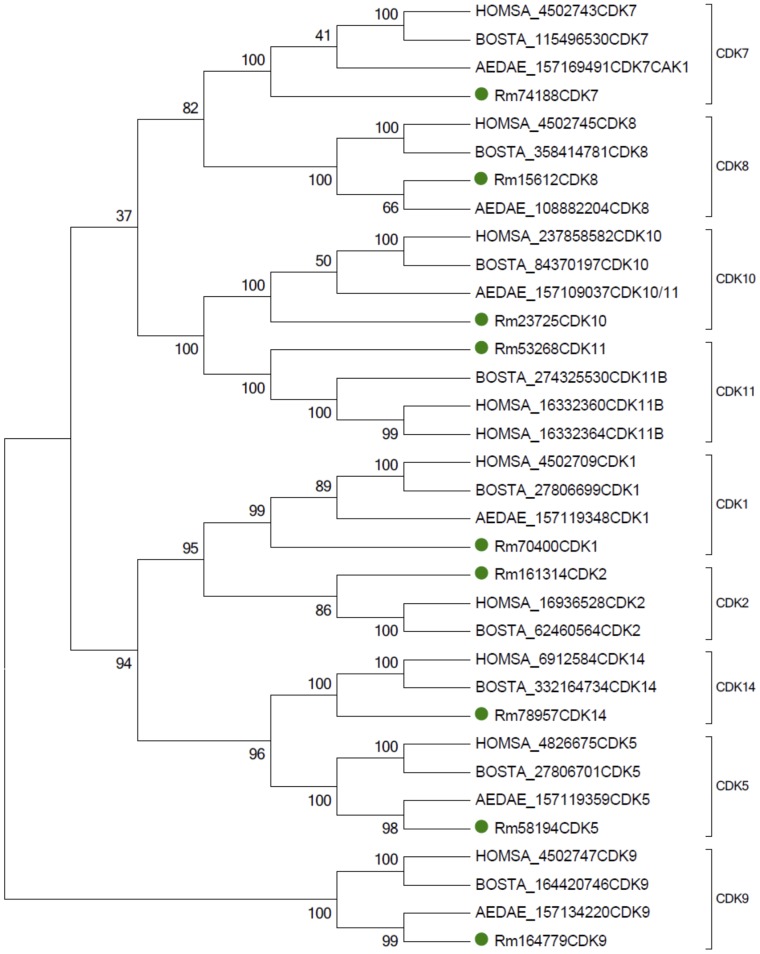
Phylogenetic analysis of *R. microplus*, *A. aegypti*, *B. taurus* and *H. sapiens* CDK sequences constructed by the neighbor-joining method using 5.1 MEGA software. Bootstrap values of 500 simulations are shown at the branches. Nine CDK isoforms found in *R. microplus* and the homologous sequences from *H. sapiens, B. taurus* and *A. aegypti* fell within specific clades corresponding to the nine CDK isoforms reported in other organisms.

A local database with all protein sequences deduced from the contigs assembled from RNA-Seq of *R. microplus* representative of the tick transcriptome was constructed (unpublished data). Blast searches for CDK-like proteins were performed in this database as described in methods. At least 9 protein sequences similar to CDK isoforms were found in *R. microplus* and tentatively named as Rm-CDK1, Rm-CDK2, Rm-CDK5, Rm-CDK7, Rm-CDK8, Rm-CDK9, Rm-CDK10, Rm-CDK11 and Rm-CDK14. Names were based on the level of sequence similarity with homologs present in HomoloGene. These CDK isoforms found in *R. microplus* and the homologous sequences from *H. sapiens, B. taurus* and *A. aegypti* are depicted in Figure 1. In all cases, the nine *R. microplus* derived sequences fell within specific clades corresponding to the nine CDK isoforms reported in other organisms. Most hematophagous arthropods use the r-strategy for reproduction, characterized with high fecundity. In the case of *R. microplus*, a single female can lay as many as 2,500 eggs. As a result, targeting the reproduction process is an attractive point of intervention in controlling this ectoparasite. From the RNA-Seq output, the number of transcripts corresponding to the *R. microplus* CDKs in various tick tissues were computed (Table 1). Rm-CDK1 and Rm-CDK10 were found to be expressed at the high levels in ovaries making them candidates for subsequent investigation.

**Table 1:** CDK-gene read counts on RNA-Seq of *R. microplus* tissues.

CDK	Ovary	Embryo	Synganglyon	Salivary gland	Fat body	Partially engorged female	Fully engorged female
**CDK1**	4503	320	158	409	523	543	101
**CDK2**	180	15	33	0	7	0	4
**CDK5**	3322	224	565	1093	567	822	127
**CDK7**	2379	92	852	222	603	372	494
**CDK8**	1272	133	532	155	184	118	125
**CDK9**	1130	40	69	58	58	56	18
**CDK10**	4844	381	605	946	1222	1176	224
**CDK11**	2759	373	492	873	430	758	218
**CDK14**	2159	47	47	202	154	157	63

CDKs orthologous proteins from *R. microplus* were identified in the available data obtained from a multi-tissue transcriptome. Messenger RNA was extracted from seven tissues of female ticks harvested at several points of development and sequenced using Illumina technology.

### Cloning and sequence analyses of Rm-CDK 1 and Rm-CDK 10 from the ovary of fully engorged tick females

**Figure 2:**
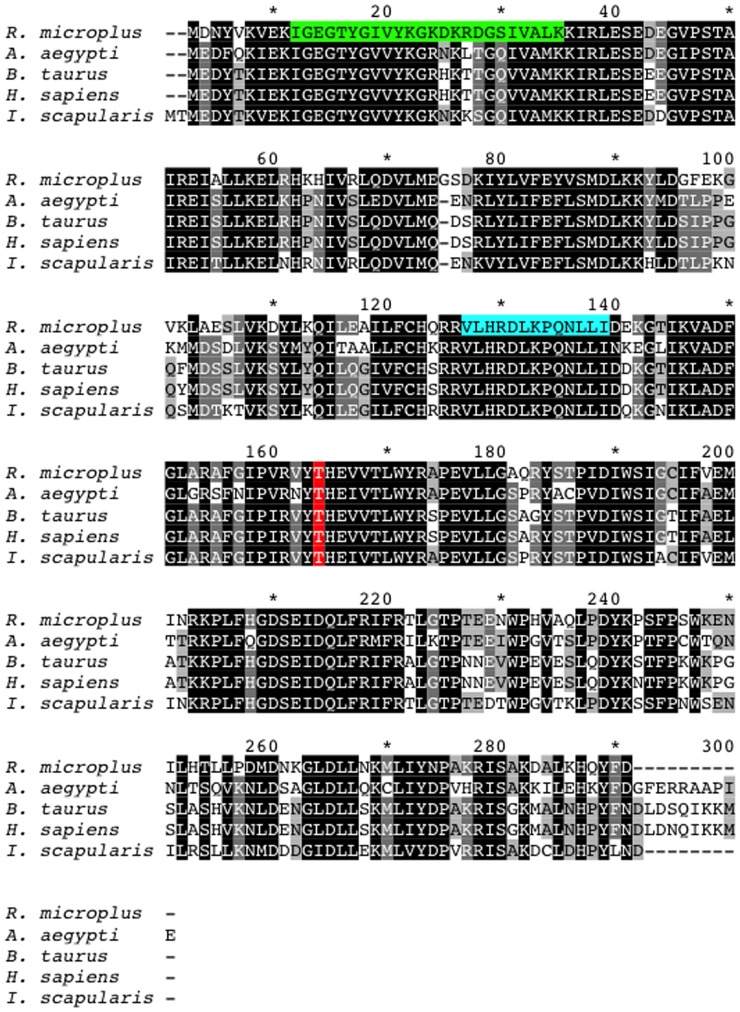
Amino acid sequence alignment of CDK1s from *R. microplus, A. aegypti, B. taurus, H. sapiens* and *I. scapularis*. Sequences were aligned using ClustalW. Conserved residues are black (100% conservation), dark grey (80% conservation), light grey (60% conservation) and no shading denotes residues with <60% conservation. Threonine residue in the activation loops is in red, protein kinases ATP-binding region signature is in green and serine/threonine protein kinases active-site signature is in blue.

**Figure 3:**
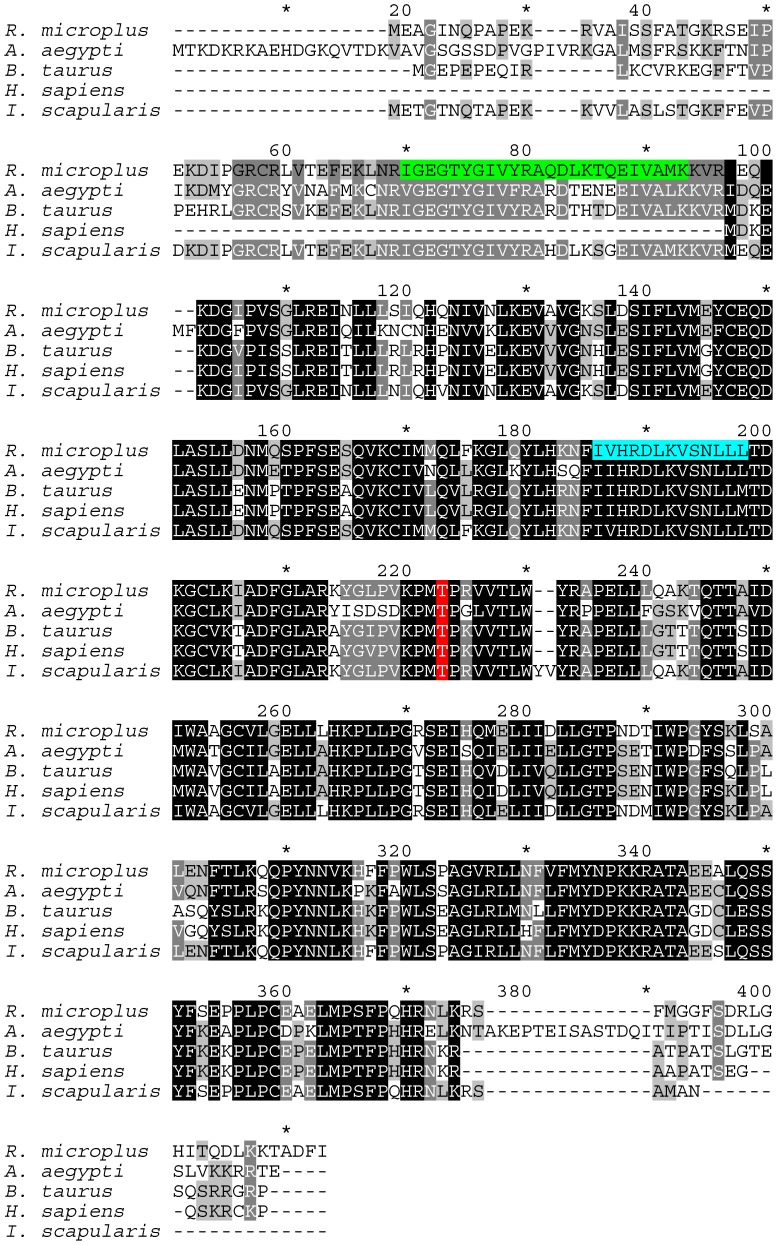
Amino acid sequence alignment of CDK10s from *R. microplus, A. aegypti, B. taurus, H. sapiens* and *I. scapularis*. Sequences were aligned using ClustalW. Conserved residues are black (100% conservation), dark grey (80% conservation), light grey (60% conservation) and no shading denotes residues with <60% conservation. Threonine residue in the activation loops is in red, protein kinases ATP-binding region signature is in green and serine/threonine protein kinases active-site signature is in blue.

An 870 bp cDNA product corresponding to Rm-CDK1 and one measuring 1122 bp cDNA product corresponding to Rm-CDK10 were amplified from the RNA of the ovary of fully engorged tick females. The obtained sequences display high similarity with CDKs of other organisms (figures 2 and 3). The predicted mature protein for Rm-CDK1 contains 289 amino acids (calculated Mw 33,456; isoelectric point 8.29) whereas Rm-CDK10 contains 373 amino acids (calculated Mw 42,290; isoelectric point 8.85). An alignment between CDK1 and CDK10 from *R*. *microplus* and those of other organisms show high residue conservation that is characteristic of proteins with basic cellular function. Specifically, CDK1 and CDK10 of *R*. *microplus* and *B. taurus* (the host of tick) showed 80 and 76% predicted amino acid similarities, respectively, (Figures 2 and 3).

### Molecular modeling studies

#### Comparative Modeling of Rm-CDK1 and Rm-CDK10

*R*. *microplus* CDK1 and CDK10 3D comparative models were constructed based on the structures of human Cyclin Dependent Kinase 2 (CDK2, PDB ID: 2v22; [26]) and the structure of human Cyclin Dependent Kinase 9 (CDK9) (PDB ID: 3mi9; [27]) with 40.51% and 63.67% of identity, respectively [28]. The 3D models were subsequently validated for the geometric integrity. Ramachandran plots revealed that over 90% of the amino acid residues are inside the allowed regions for both comparative models (Figures S3 and S4).

#### Identification of functional motifs in CDK1 and CDK10 from *R. microplus*

**Figure 4:**
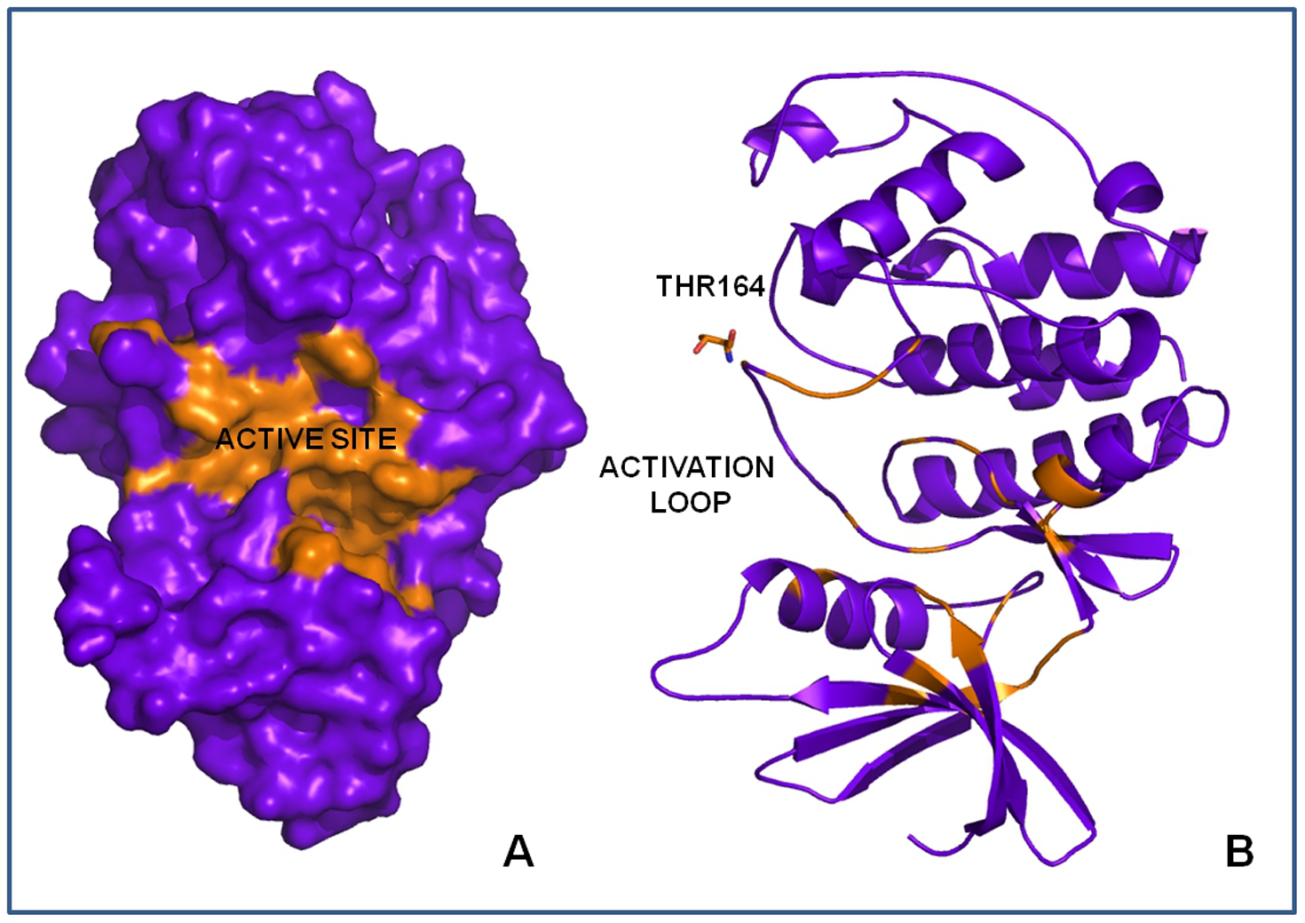
Structure of the comparative model of Rm-CDK1 showing the ATP binding Site (active site) (A) and detail of the activation loop with the putative phosphorylating residue Thr164 (B).

**Figure 5:**
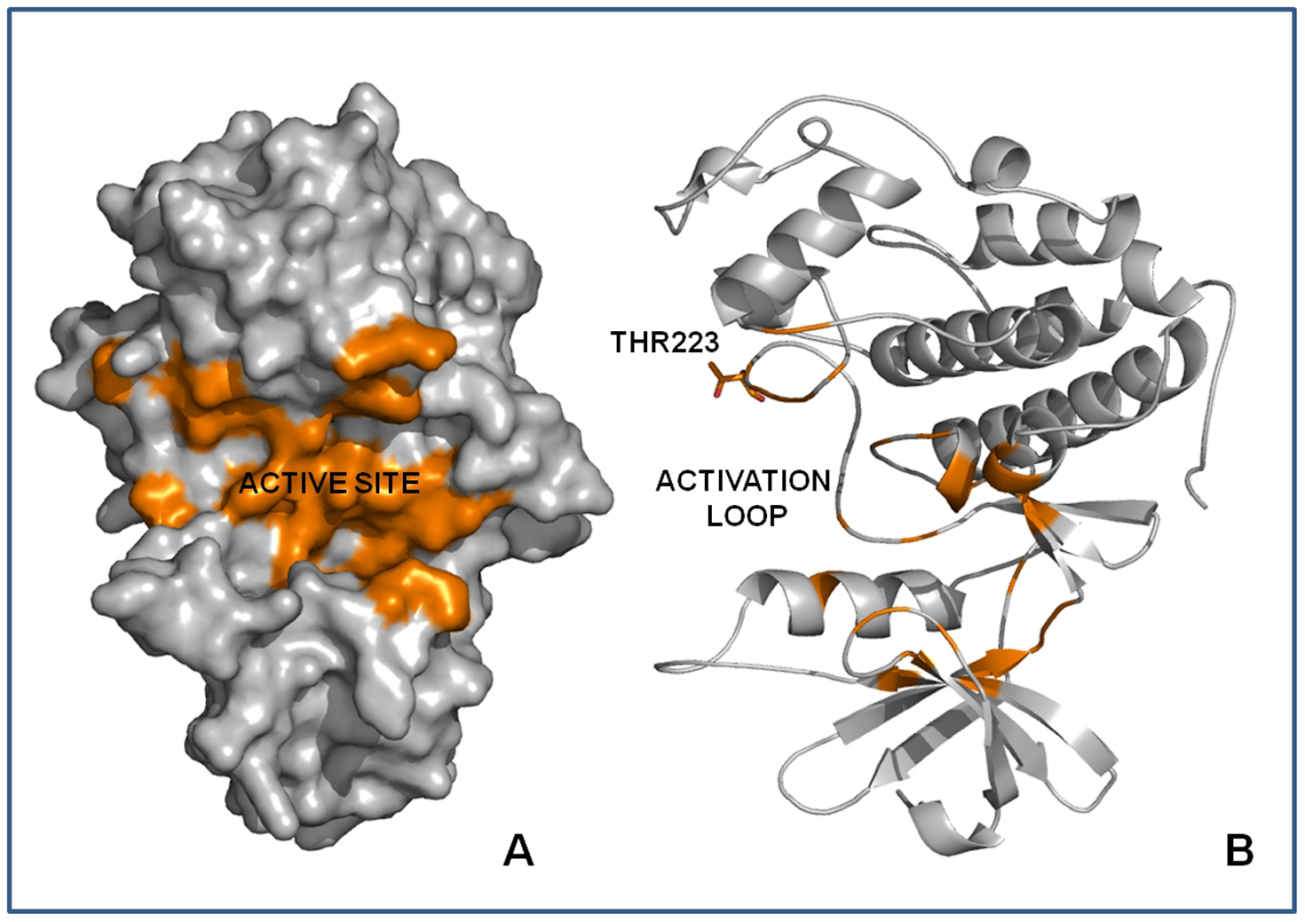
Structure of the comparative model of Rm-CDK10 showing the ATP binding Site (active site) (A) and detail of the activation loop with the putative phosphorylating residue Thr223 (B).

The ATP binding pocket, the active sites of Rm-CDK1 and Rm-CDK10 and the threonine residues in the activation loops of CDK1 (Thr164) and CDK10 (Thr223) were identified in a 3D model (Figures 4 and 5), supporting the presence of a kinase motif for these proteins. Furthermore, the Caspase 3/7 recognition motif, with the consensus sequence and an aspartic acid on the penultimate position that is essential for biological function of the CDKs, was present in these sequences. In the comparative 3D model of Rm-CDK1, two other motifs, opposite to the ATP binding site were identified (Figure S5). The motif DKRGD contains the amino acids Asp25, Lys26, Arg27, Asp28 and Gly29 in the *N*-terminus of the protein and the SAKDA sequence, with the amino acid residues Ser280, Ala281, Lys282, Asp283 and Ala284, in the *C*-terminus of the protein. In the structure of Rm-CDK10, only a SLLDN motif for caspase 3–7 located near the ATP binding site was found, with the amino acid residues Ser153, Leu154, Leu155, Asp156 and Asn157 (Figure S6).

#### Simulations of roscovitine docking with Rm-CDK1 and Rm-CDK10

**Figure 6:**
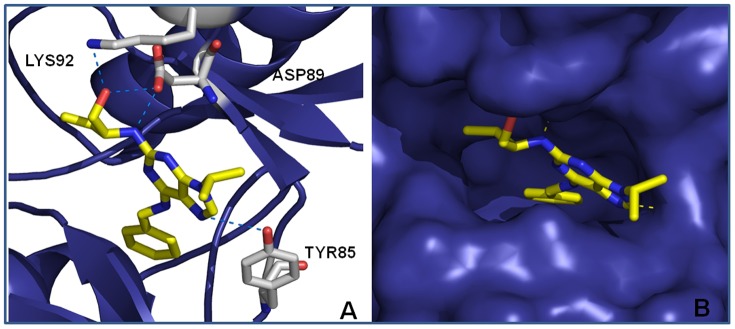
A, B: Top pose obtained by docking of roscovitine with Rm-CDK1 comparative model (yellow carbon atoms). Hydrogen atoms have been omitted for a better view. Hydrogen bonds are depicted in blue dashed lines.

**Figure 7:**
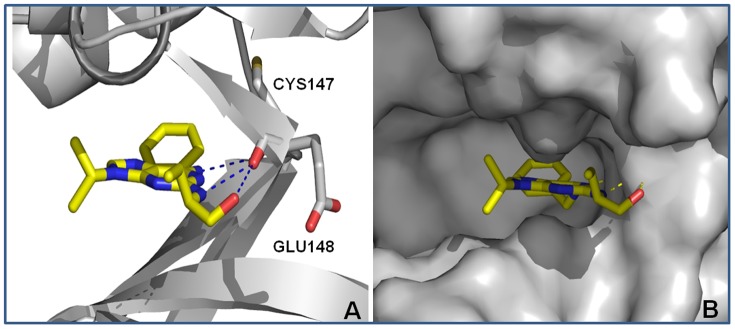
A, B: Top pose obtained by docking of roscovitine with Rm-CDK10 comparative model (yellow carbon atoms). Hydrogen atoms have been omitted for a better view. Hydrogen bonds are depicted in blue dashed lines.

Using the modeled 3D structures of Rm-CDKs1 and 10, a simulated docking of roscovitine on the two proteins suggested a stable interaction (Figures 6 A–B for Rm-CDK1 and Figures 7 A–B for Rm-CDK10). A putative interaction of roscovitine in the ATP binding site was observed in both Rm-CDKs. In both cases, the *N*-benzyl ring is oriented away from the solvent accessible surface and onto the ATP binding pocket (Figures 6B and 7B). In Rm-CDK1, one of the nitrogen atoms of the purine ring of roscovitine hydrogen bonds to Tyr85 while the nitrogen and the oxygen atoms of the hydroxyethylamino moiety perform ion-dipole interactions with Asp89 and Lys92, respectively (Figure 6A). Concerning Rm-CDK10, there are hydrogen bonds involving the nitrogen atom of the purine ring and the nitrogen and oxygen atoms on the hydroxyethylamino moiety of roscovitine on one side, and the backbone oxygen of Cys147 and Glu148 (Figure 7A). When this *in silico* analysis was compared with experimental observations that show the binding of roscovitine to human CDK2 (PDB ID: 2A4L; [29], for example, the putative ATP binding sites of our 3D models adopt a more closed conformation, and impairs the interaction with other proteins (Figure S7). Furthermore, superimposition of Rm-CDKs1 and 10 models with the crystal structure of human CDK2 (Figure S8) shows different binding modes, in which roscovitine performs more interactions than it is observed experimentally with human CDK2 as stated previously. In the crystal structure, roscovitine hydrogen bonds to a water molecule and to the oxygen atom of the carbonyl group of Leu83 (Figure S7). Thus, despite amino acid residue conservation in the ATP binding site, differences in Rm-CDKs1 and 10 are enough to suggest different binding modes for Roscovitine (Figures S8 and S9). However, it is not possible to rule out the idea that conformational differences may have been inherited by the templates used for modeling in the CPHModels server. For instance, the careful analysis of the amino acid residues surrounding Roscovitine binding site in human CDK2 shows, in general, the same corresponding amino acid residues in the Rm-CDK1 model. One noticeable difference is the inverted conformation of the long side chain of Lys89 in human CDK2 *vs*. Lys92 in Rm-CDK1 (Figure S9), which is involved in an ion-dipole interaction to Roscovitine in our Rm-CDK1 model and may have oriented a different binding mode compared to human CDK2. Another amino acid substitution worth mentioning is Phe82 in CDK2, which has Tyr85 as the corresponding amino acid in Rm-CDK1 model and is involved in hydrogen bonding with Roscovitine in our model. Further differences have been observed only in regions that are distant from the active site or the activation loop (data not labeled). Nevertheless, when we compare amino acid residues from Rm-CDK10 and human CDK2 (Figure S8), the differences are not only conformational but also in the volume and/or physicochemical characteristics of the amino acids in the active site. For instance, Glu148, which is involved in a hydrogen bond with Roscovitine in the Rm-CDK10 model has His84 as the corresponding amino acid in human CDK2. Also, Cys147 in Rm-CDK10 has been replaced by Leu83 in human CDK2 and, at the surface of the active site, Arg269 in Rm-CDK10 has been replaced by Asp206 in human CDK2, which provides a totally different electrostatic environment to the active site. These data support the fact that, overall, Rm-CDK10 is less conserved in the active site than Rm-CDK1 when compared to human CDK2. Finally, another point for discussion is the fact that water molecules have not been modeled or included in the docking studies, though they may have an important role in the binding affinity of Roscovitine.

### 3.4. Viability assay and inhibitory effect of roscovitine on growth of BME26 Cells

**Figure 8:**
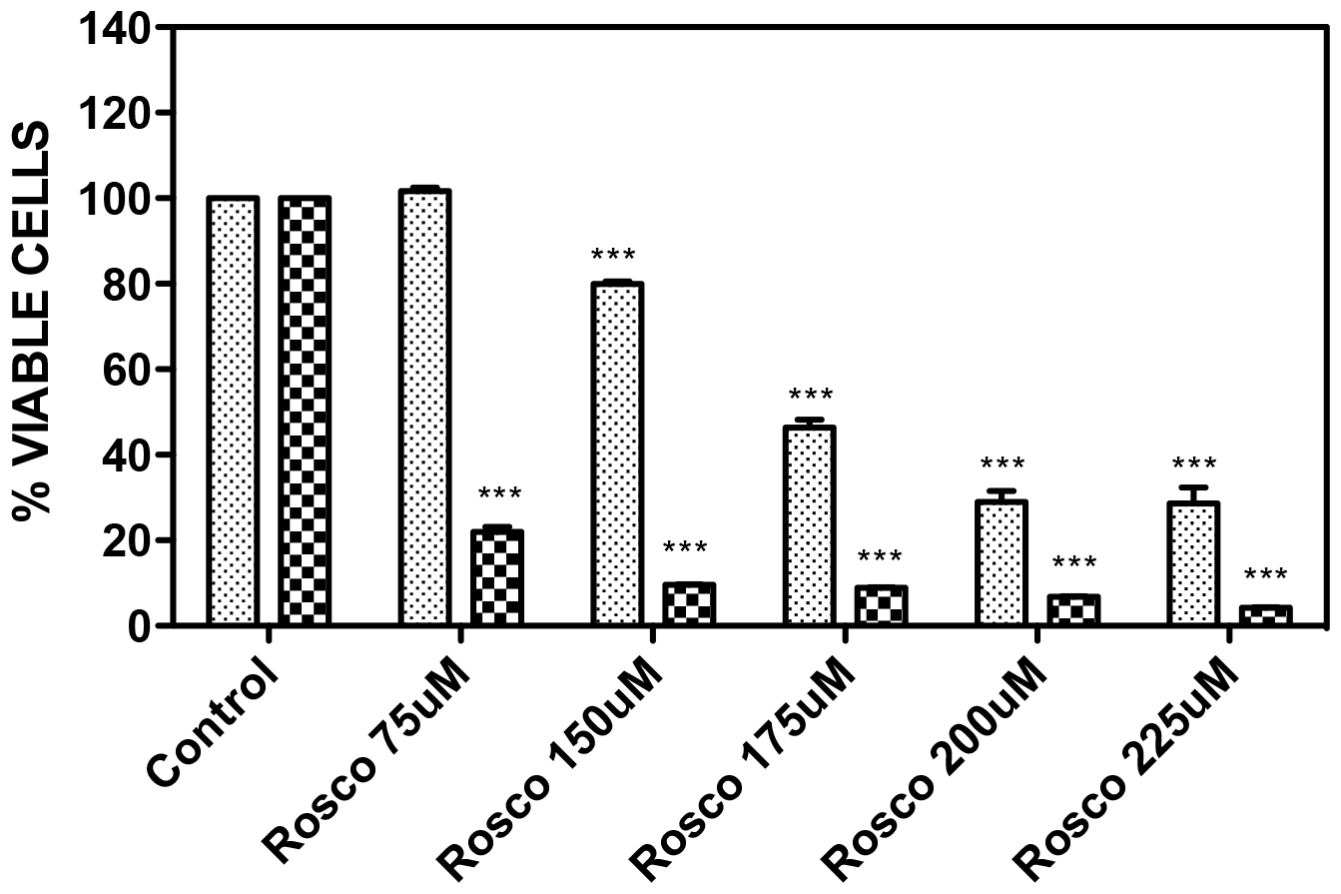
MTT viability assay of BME26 cells. BME26 cells were incubated with different concentrations of roscovitine (a CDK inhibitor) or DMSO 0.1% (control) for 24 h (gray bar) and 48 h (grid bar) and then cell viability was determined by the MTT assay. After incubation the medium was removed and added MTT for 2 h. Reaction was read in spectrophotometer at 570 nm. Graph represents three independent experiments in triplicate. On all tested concentrations, with the exception of roscovitine 75 µM for 24 h, values of roscovitine groups were significantly different (One-way analysis of variance—ANOVA, *p*<0.05) as compared to the control groups.

BME26 cells were incubated with different concentrations of roscovitine for 24 h or 48 h to assess the effect of the inhibitor on the cells. Following 24 h incubation, the viability of the cells decreased in a concentration-dependent manner. The cell number was reduced to 80% (150 µm), 45% (175 µm), 30% (200 µm) and 26% (225 µm) relative to the control cells, which contained only DMSO (Figure 8). The cytotoxic effect of roscovitine on BME26 cells was further pronounced after 48 h of incubation, with cell viability dropping to 22% (75 µm), 9.6% (150 µm), 9% (175 µm), 7% (200 µm), and 4% (225 µm) relative to the control group (Figure 8).

**Figure 9:**
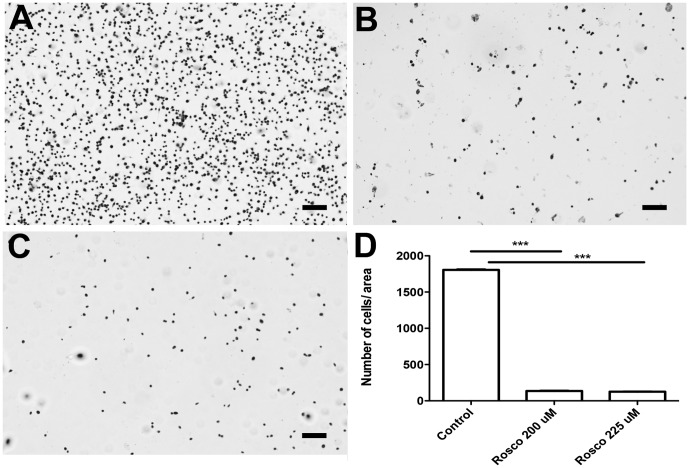
Hematoxylin-eosin staining of the BME26 cell line upon exposure to roscovitine BME26 cells after 24 h of incubation with different concentrations of roscovitine were stained with Hematoxylin and Eosin. A) Control with addition of 0.1% DMSO; B) rosco 150 µM; C) rosco 200 µM. The cells were observed under a light microscope. Magnification: 10X. The scale corresponds to 100 µm. Pictures are representative of 3 independent experiments in triplicates. D) Graphic represents the number of cells in each treatment quantified by image J in three different fields. On all tested concentrations, values of roscovitine groups were significantly different (One-way analysis of variance—ANOVA, *p*<0.05) as compared to the control groups.

The susceptibility of embryonic tick cells to roscovitine was further confirmed by hematoxylin and eosin (HE) staining that assess the number of cells per field by marking the cell nucleus. As evident from Figure 9, there was significant decline in the number of cells per field following treatment with roscovitine concentrations higher than 150 µM. Statistical significance of inhibitory effect was analyzed with One-way analysis of variance—ANOVA. Results are expressed, as mean ±S.D. p values ≤0.05 were considered statistically significant.

### Transcription of Rm-CDKs in BME26

**Figure 10:**
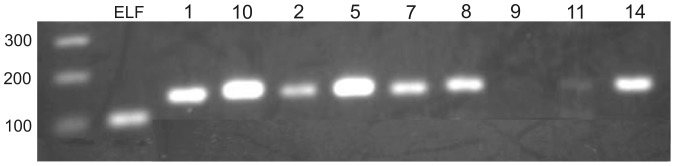
Identification of Rm-CDKs in BME26 cells. RT-PCR of BME26 cells showing the transcription of Rm-CDK1, 2, 5, 7, 8, 10, 11 and 14 in BME26 cells. ELF1A was used as a positive control. WM – weight marker; ELF – constitutive gene (108 bp); 1 – CDK1 (148 bp); 10 – CDK10 (155 bp); 2 – CDK2 (149 bp); 5 – CDK5 (151 bp); 7 – CDK7 (147 bp); 8 – CDK8 (152 bp); 9 – CDK9 (155 bp); 11 – CDK11 (150 bp); 14 – CDK14 (149 bp).

RT-PCR analysis showed that Rm-CDK1, 2, 5, 7, 8, 10, 11 and 14 were transcribed in BME26 cells. Rm-CDK9 transcription was not detectable in BME26 cells (Figure 10). In general, RT-PCR results of BME26 cells are in agreement with the RNA-Seq analysis of embryo (Table 1).

## Discussion

Cyclin-dependent protein kinases (CDKs) are responsible for controlling eukaryotic cell division cycle [30]. In complex cell cycles, they also integrate extracellular and intracellular signals to regulate cell cycle events in response to signals from the environment or cellular damage and to control the transition to subsequent phases of the cycle [30]. CDKs are small serine/threonine protein kinases that require association with a cyclin subunit for their activation [31]. CDKs are the catalytic subunits of heterodimeric complexes that are activated or inactivated at specific stages of the cell cycle triggering the next cell cycle events [8]. Each catalytic CDK subunit can associate with different cyclins, and this determines which one is phosphorylated by the cyclin-CDK complex [32].

Roscovitine (Rosco) is a purine derivative that inhibits CDKs by binding to the catalytic domain of the CDK molecule in the place of ATP and blocks the transfer of a phosphate group to the substrate [33]. As an inhibitor of CDK, Rosco is widely used as an anti-cancer drug and is capable of inducing apoptosis in isolated B-CLL cells by caspase activation and modulation of bcl-2 family proteins [34].

In the current study the tick embryo cell line BME26 was incubated for a period of between 24 and 48 h in medium supplemented with roscovitine. The observed dose-dependent decrease in cell viability was consistent with past findings with rabbit retinal pigment epithelial cells [35], glioma cells [36], polymorphonuclear cell [37] and tumor cell lines [38], [39]. The data obtained with different concentrations of roscovitine at and incubation times, in MTT assay and visual counting, suggest that it induces cell death instead of reducing cellular metabolic activity [40].

*R. microplus* CDKs may present suitable targets whose blockade may interfere with the vital developmental processes of this tick, such as egg production and embryogenesis. Ticks, like many other hematophagous arthropods have high fecundity, and a female tick may lay up to 2,500 eggs. The transcriptome analysis study conducted in the present study showed that Rm-CDKs 1 and 10 are highly upregulated in the ovaries. It seems that the genes coding for these proteins are highly expressed in the adult female tick and that these motherly expressed genes could be crucial to embryo development and consequently to the biological success of this species reproduction. Consequently, cDNAs corresponding to the ORFs of the two CKDs were cloned and the deduced protein sequences utilized *in silico* structural analyses. BLAST searches and Eukaryotic Linear Motif Resource simulation indicated the presence of ATP-binding functional motifs and Caspase 3–7 recognition. This is supported by docking models that showed roscovitine could inhibit both Rm-CDKs 1 and 10 by binding it to the putative ATP-binding site. This interaction between roscovitine and CDKs 1 and 10 may extend to other *R*. *microplus* CDKs owing to the largely conserved kinase active site but this is yet to be evaluated.

Roscovitine can induce apoptosis by activating mitochondrial caspase pathway or modulating the expression of the Bcl-2 family member [41], [42]. All *R. microplus* CDKs were obtained from multiple tick tissues through RNA-sequencing. The analysis of these protein sequences on the eukaryotic Linear Motif application that predicts protein functional sites showed that Rm-CDK1 has 2 motifs for caspase 3–7 and Rm-CDK10 has one motif for caspase 3–7 and these motifs are located at different positions suggesting functional non-redundancy. The presence of these motifs reinforces the suggestion of a role for *R. microplus* CDKs in the cell cycle similar to mammal CDKs, in which caspases 3 and 7 have a critical role in mediating the downstream mitochondrial events of the intrinsic pathway in apoptosis [43]–[45]. CDKs are important regulators of the cell cycle and are also known as cycle checkpoint. During the process of maturation of the mammalian oocyte, meiosis resumption occurs so the oocyte can mature and be fertilized. This process is regulated by several proteins, among them the CDKs. The regulation of CDK activity is directly related to the process of maturation and the production of blastocysts [46]–[48]. The addition of roscovitine, the CDK inhibitor, to the culture medium delays the completion of meiotic maturation of the bovine oocytes resulting in a higher blastocyst formation [12]. Farrell et al. [49] demonstrated that the down-regulation of CDK1 activity triggers the onset of late-replicating DNA and an increase in S-phase length in *Drosophila* embryos. Extrapolating from this observation, it is possible that *R. microplus* CDKs may participate in egg development. The increased expression of the CDKs in the ovaries from *R. microplus* suggests that, as in mammals, these proteins could participate in egg development. The participation of CDKs in the regulation of cell cycle and the formation of the embryo has already been reported in other invertebrates such as *C. elegans* [50] and *Drosophila* [51], [52], and the parasite *Leishmania donovani* [53].

This is the first report to demonstrate a cell cycle checkpoint protein in arachnids and the reversal of its functions with an inhibitor. Overall, these results suggest that CDKs are candidate targets for blockade with roscovitine and related compounds, which may present an alternative strategy for designing drugs against *R. microplus* that target both oogenesis and embryogenesis processes.

## Supporting Information

Figure S1 FASTA-formatted file containing multiple sequences of representative CDKs.
(FAS)

Figure S2 Phylogenetic analysis with representatives of the 20 types of CDK found in HomoloGene and ticks (*R. microplus* and *I. scapularis*) CDKs constructed by the neighbor-joining method using 5.1 MEGA software. Bootstrap values of 500 simulations are shown at the branches.
(PDF)

Figure S3 Ramachandran Plot of the comparative model of Rm-CDK1 from *R*. *microplus*.
(TIF)

Figure S4 Ramachandran Plot of the comparative model of Rm-CDK10 from *R*. *microplus*.
(TIF)

Figure S5 Structure of Rm-CDK1 from *R. microplus* obtained by comparative modeling (A). The *motifs* for Caspase 3–7 SAKDA and DKRGD identified in the Eukariotic Linear Motif Resource are shown in the detail (B and C). SAKDA and DKRGD binding surfaces along with the ATP binding pocket (D).
(TIF)

Figure S6 Structure of Rm-CDK10 from *R. microplus* obtained by comparative modeling (A). The *motif* for Caspase 3–7 SLLDN identified in the Eukariotic Linear Motif Resource is shown in the detail (B). SLLDN binding surfaces along with ATP binding pocket (C).
(TIF)

Figure S7 Crystal Structure of roscovitine Bound to Human CDK2. Hydrogen atoms have been omitted for a better view. Hydrogen bonds are depicted in blue dashed lines.
(TIF)

Figure S8 Superimposition of the top scored poses of roscovitine (yellow carbon atoms) obtained by docking with Rm-CDK1 (A) and Rm-CDK10 (B) models and roscovitine (magenta carbon atoms) co-crystallized with human CDK2 (cyan). Hydrogen bonds are depicted in blue dashed lines.
(TIF)

Figure S9 Representative substitutions of amino acid residues in roscovitine binding site in Rm-CDK1 (A, deep blue) and Rm-CDK10 (B, light gray) models compared to human CDK2 (cyan).
(TIF)

Table S1 The Rm-CDK gene specific primers used in RT-PCR analysis.
(PDF)
