# Supplementary figures and images for: Identification and Structural-Functional Analysis of Cyclin-Dependent Kinases of the Cattle Tick Rhipicephalus (Boophilus) microplus
Source: PLoS One. 2013 Oct 11;8(10):e76128. doi: 10.1371/journal.pone.0076128 (PMC3795742; doi:10.1371/journal.pone.0076128)

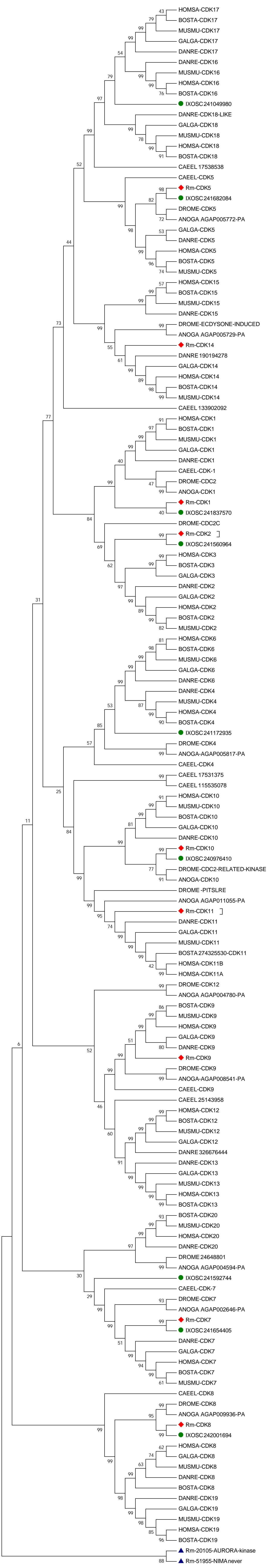

Supplement: Figure S2 — Phylogenetic analysis with representatives of the 20 types of CDK found in HomoloGene and ticks (R. microplus and I. scapularis) CDKs constructed by the neighbor-joining method using 5.1 MEGA software. Bootstrap values of 500 simulations are shown at the branches. (PDF) [file pone.0076128.s002.pdf]

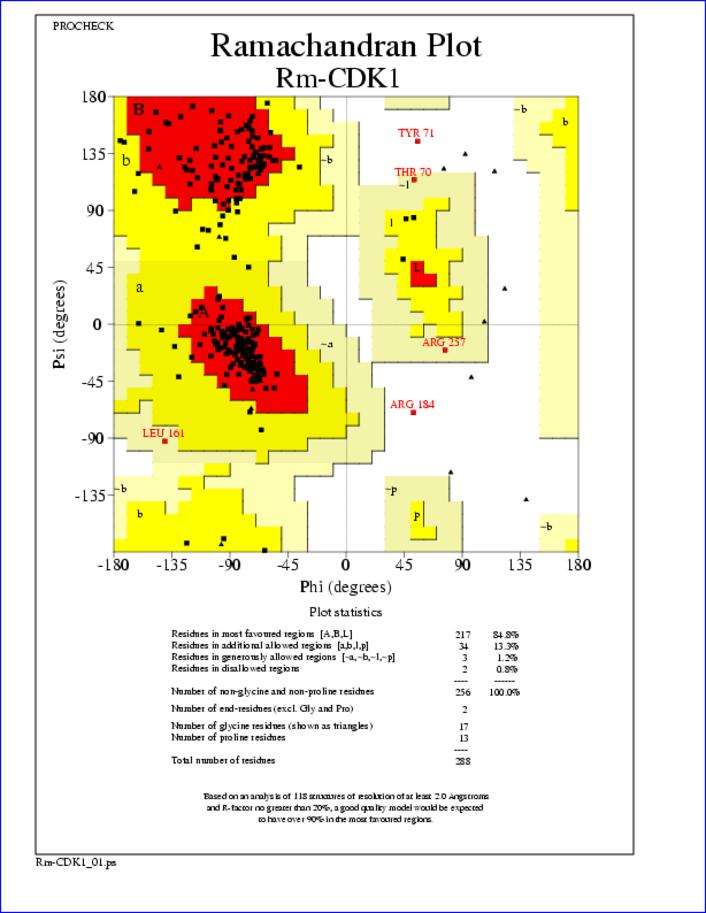

Supplement: Figure S3 — Ramachandran Plot of the comparative model of Rm-CDK1 from R. microplus. (TIF) [file pone.0076128.s003.tif]

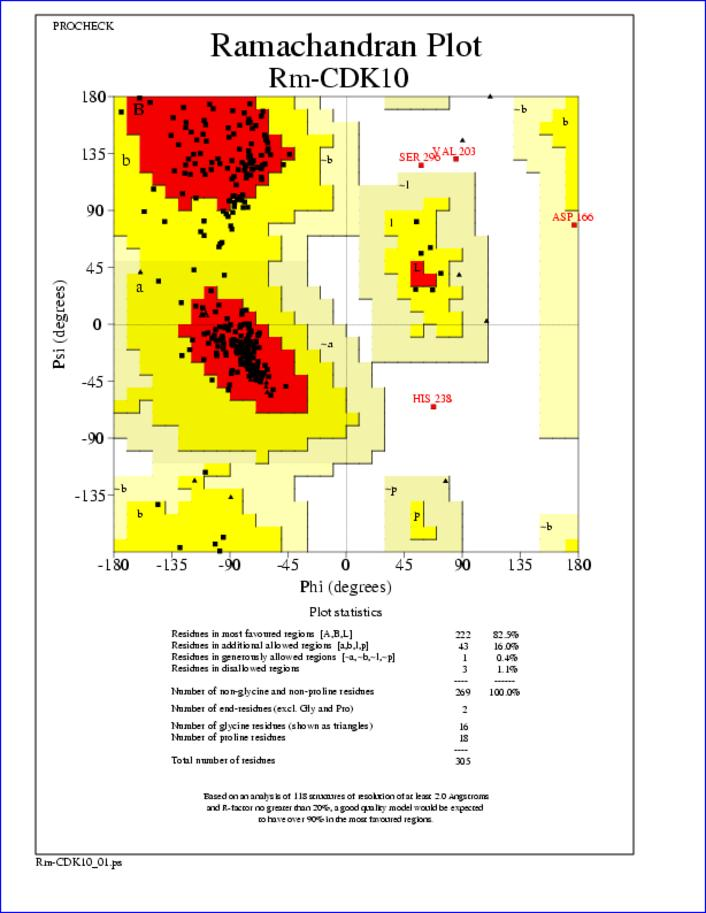

Supplement: Figure S4 — Ramachandran Plot of the comparative model of Rm-CDK10 from R. microplus. (TIF) [file pone.0076128.s004.tif]

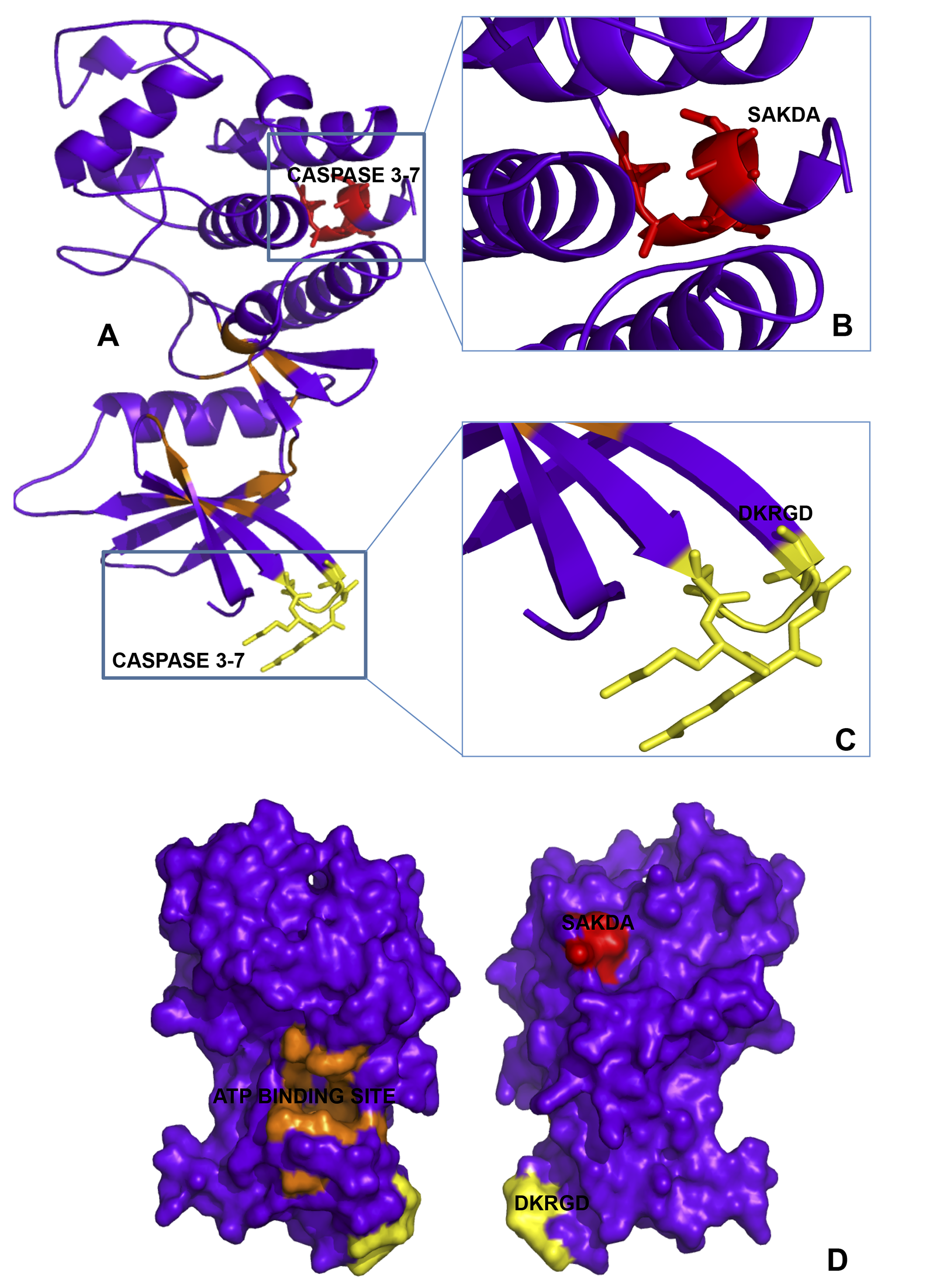

Supplement: Figure S5 — Structure of Rm-CDK1 from R. microplus obtained by comparative modeling (A). The motifs for Caspase 3–7 SAKDA and DKRGD identified in the Eukariotic Linear Motif Resource are shown in the detail (B and C). SAKDA and DKRGD binding surfaces along with the ATP binding pocket (D). (TIF) [file pone.0076128.s005.tif]

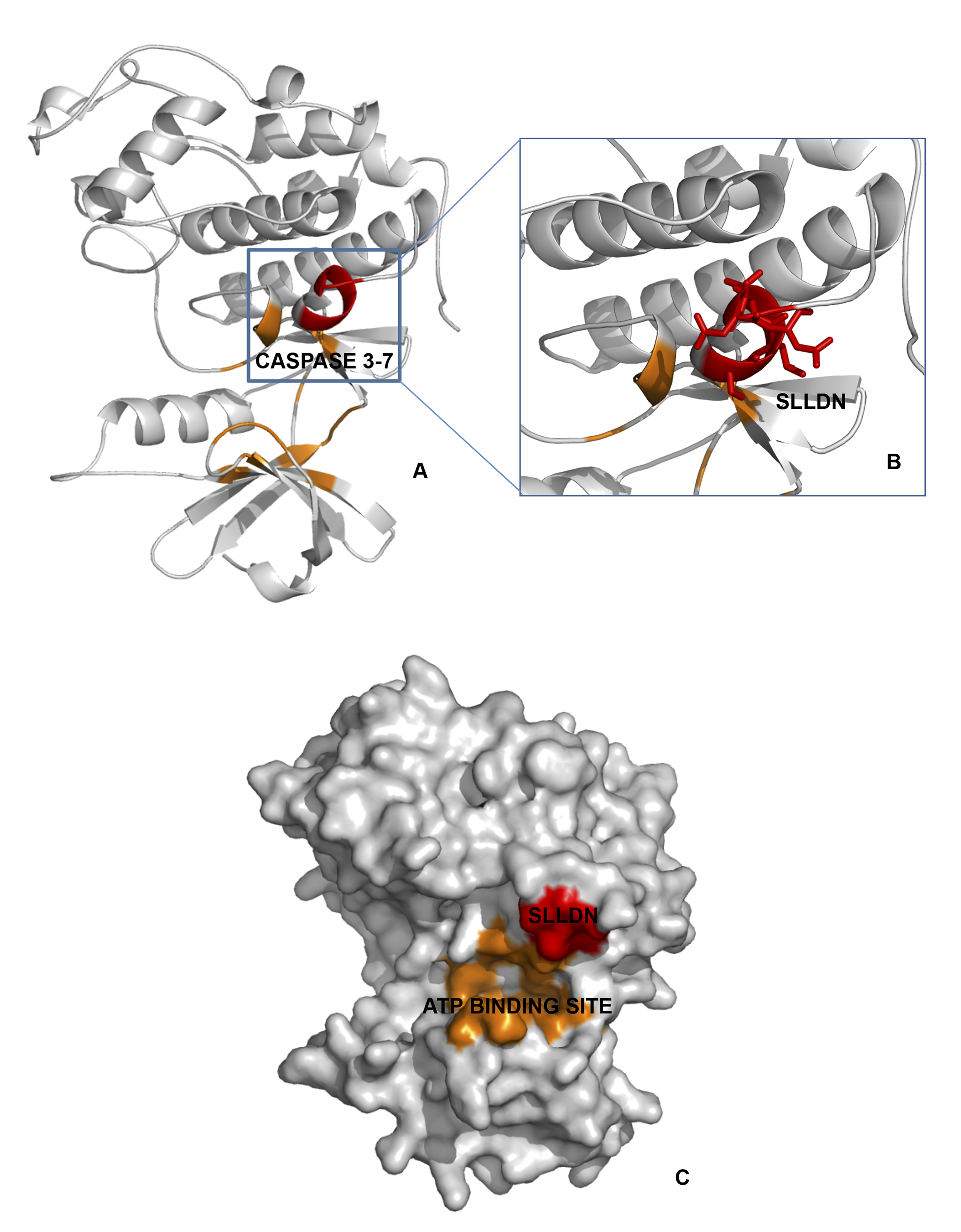

Supplement: Figure S6 — Structure of Rm-CDK10 from R. microplus obtained by comparative modeling (A). The motif for Caspase 3–7 SLLDN identified in the Eukariotic Linear Motif Resource is shown in the detail (B). SLLDN binding surfaces along with ATP binding pocket (C). (TIF) [file pone.0076128.s006.tif]

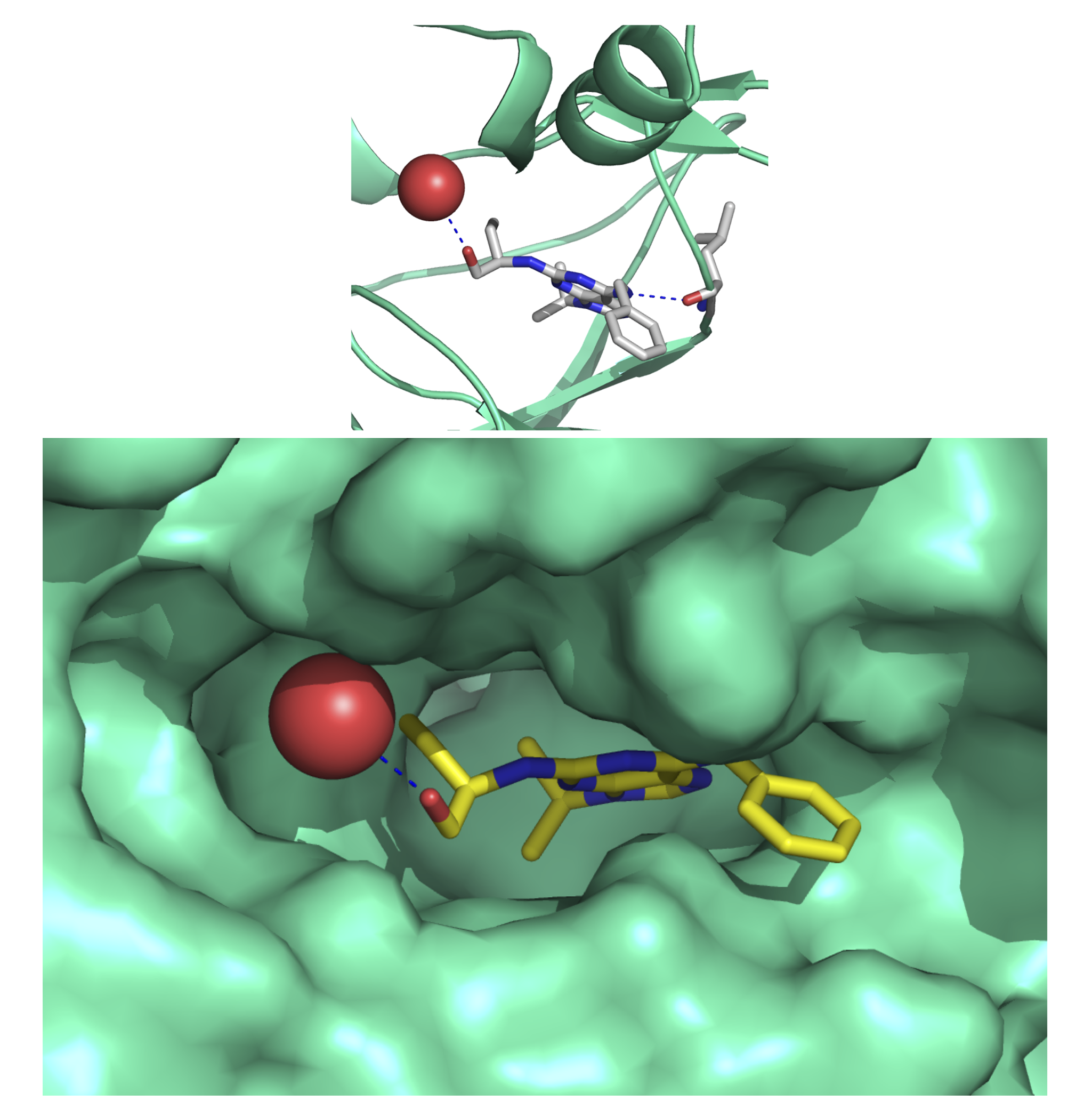

Supplement: Figure S7 — Crystal Structure of roscovitine Bound to Human CDK2. Hydrogen atoms have been omitted for a better view. Hydrogen bonds are depicted in blue dashed lines. (TIF) [file pone.0076128.s007.tif]

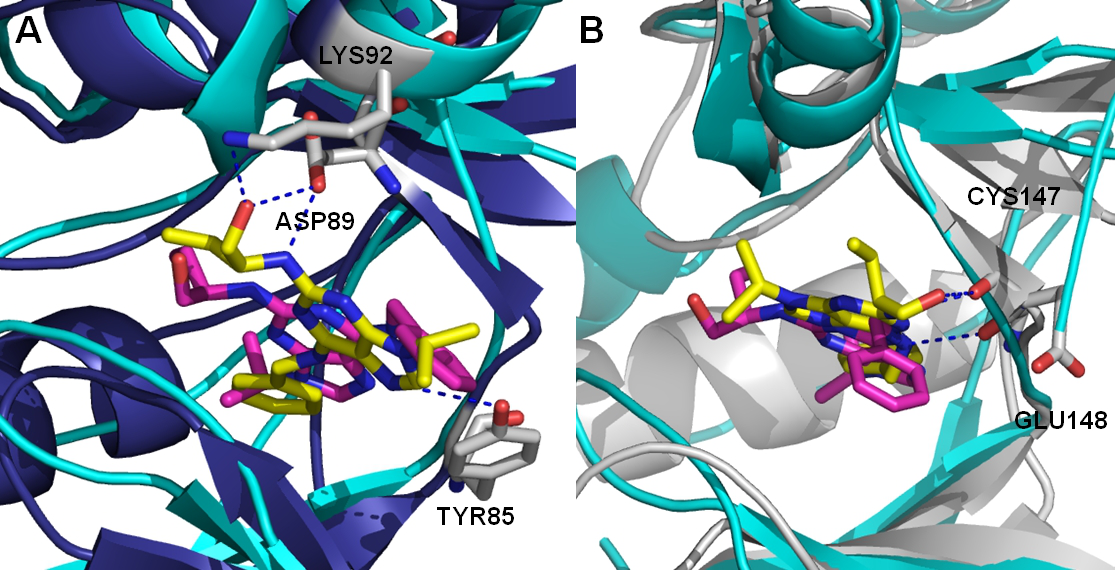

Supplement: Figure S8 — Superimposition of the top scored poses of roscovitine (yellow carbon atoms) obtained by docking with Rm-CDK1 (A) and Rm-CDK10 (B) models and roscovitine (magenta carbon atoms) co-crystallized with human CDK2 (cyan). Hydrogen bonds are depicted in blue dashed lines. (TIF) [file pone.0076128.s008.tif]

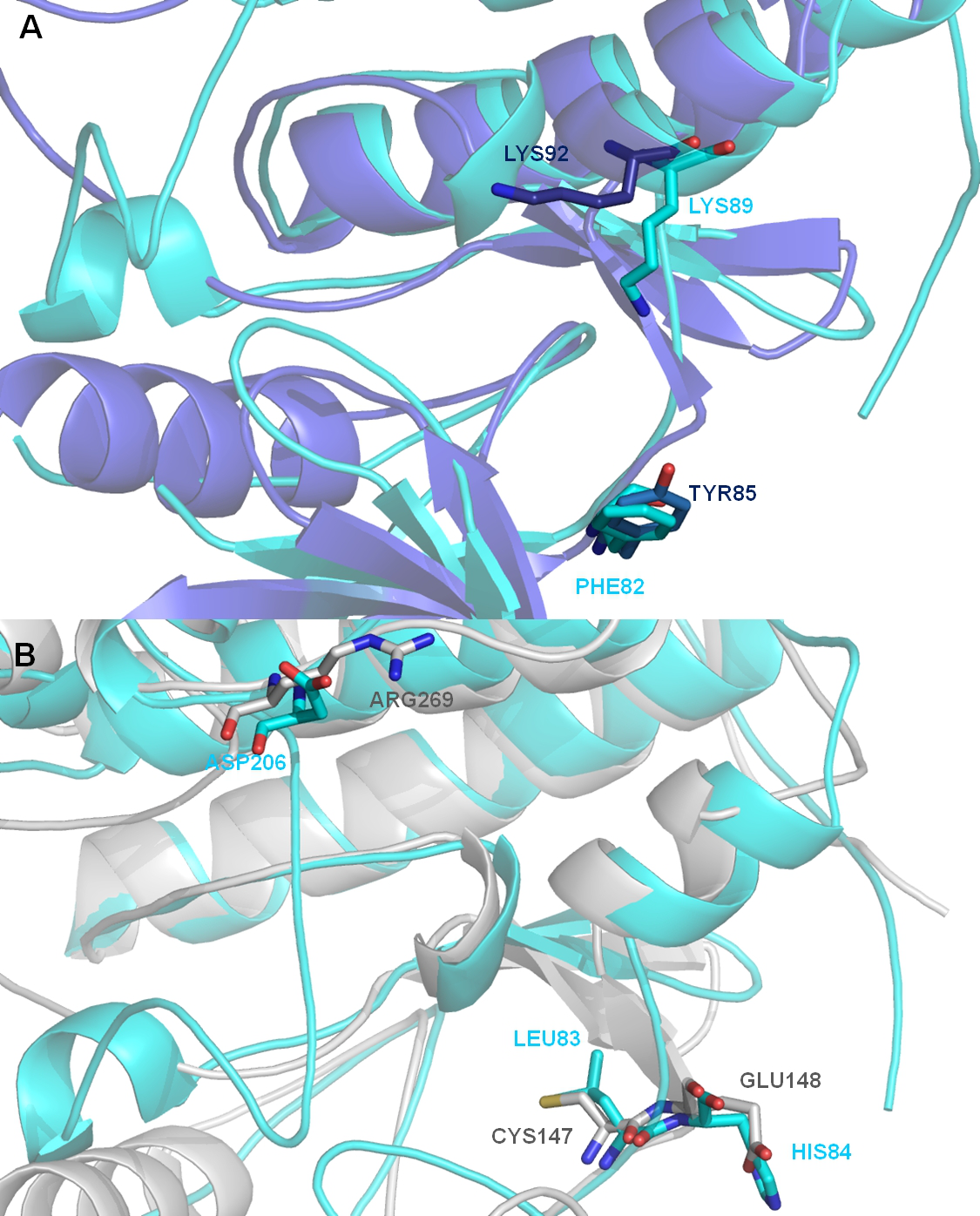

Supplement: Figure S9 — Representative substitutions of amino acid residues in roscovitine binding site in Rm-CDK1 (A, deep blue) and Rm-CDK10 (B, light gray) models compared to human CDK2 (cyan). (TIF) [file pone.0076128.s009.tif]
