# Supplementary material for: Identification and Structural-Functional Analysis of Cyclin-Dependent Kinases of the Cattle Tick Rhipicephalus (Boophilus) microplus
Source: PLoS One. 2013 Oct 11;8(10):e76128. doi: 10.1371/journal.pone.0076128 (PMC3795742; doi:10.1371/journal.pone.0076128)
Supplement: Table S1 — The Rm-CDK gene specific primers used in RT-PCR analysis. (PDF) [file pone.0076128.s010.pdf]

Supporting Table 1- The Rm-CDK gene specific primers used in RT-PCR analysis.

ID	Primer forward (5' -3')	Primer reverse (5' -3')
CDK1	CGGGCGAAAACGAACGTCTT	GTGGCGGCCTTGCAATATCC
CDK2	CGCTCAGTGCGAAGTTCCAGAA	GGCAGTGCTTGGTACACCTTCG
CDK5	CGATGACGACGACGAGGGAGTA	CAGATCCTGGTCACAGTGCTCGA
CDK7	GATACGAAAAGATCGAGTTCCTCGG	ACGCTGTCCGGTTAATGCCAT
CDK8	TTCAAAGTCAAGACGGCTAGTGTGC	TCAATTTGCTTCAGTGCATAATCCC
CDK9	GGTGATGCAACAGCTTCTCAATGG	CCGTTTTACTCAGGGAGAAGGCC
CDK10	CGATGCCGGTTAGTTACAGAGTTCG	CCACTGACAGGAATGCCATCTTTT
CDK11	CAGATGAGATTGTGGCTCTGAAACG	CCATGTTGCTTCCCACCACTATCT
CDK14	TCTCCGTGAGCAGTAAGAACTGGAA	GATCACCTCCTTGGACAGCGAAA
ELF1A	CGTCTACAAGATTGGTGGCATT	CTCAGTGGTCAGGTTGGCAG
